# Triethyl­ammonium tetra­chlorido(pyridine-2-carboxyl­ato-κ^2^ *N*,*O*)stannate(IV)

**DOI:** 10.1107/S1600536811005460

**Published:** 2011-02-19

**Authors:** Ezzatollah Najafi, Mostafa M. Amini, Seik Weng Ng

**Affiliations:** a Department of Chemistry, General Campus, Shahid Beheshti University, Tehran 1983963113, Iran; b Department of Chemistry, University of Malaya, 50603 Kuala Lumpur, Malaysia

## Abstract

The cation and the anion in the title salt, (C_6_H_16_N)[SnCl_4_(C_6_H_4_NO_2_)], are linked by an N—H⋯O hydrogen bond. The Sn^IV^ atom in the stannate anion is chelated by the pyridine-2-carboxyl­ate group and exists in a *cis*-SnCl_4_NO octa­hedral geometry. The cation is disordered over two positions in a 0.564 (1):0.436 (1) ratio.

## Related literature

For another ammonium tetra­chlorido(pyridine-2-carboxyl­ato)stannate, see: Najafi *et al.* (2011 ▶).
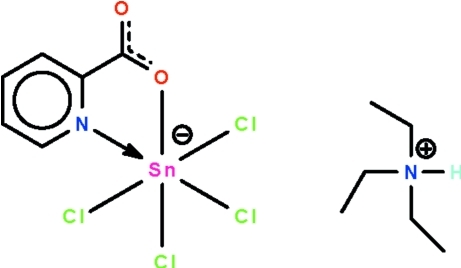


## Experimental

### 

#### Crystal data

(C_6_H_16_N)[SnCl_4_(C_6_H_4_NO_2_)]
*M* *_r_* = 484.79
Monoclinic, 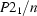

*a* = 11.6310 (7) Å
*b* = 10.4912 (6) Å
*c* = 16.4452 (9) Å
β = 109.672 (1)°
*V* = 1889.57 (19) Å^3^
*Z* = 4
Mo *K*α radiation
μ = 1.92 mm^−1^
*T* = 100 K
0.30 × 0.25 × 0.05 mm

#### Data collection

Bruker SMART APEX diffractometer
Absorption correction: multi-scan (*SADABS*; Sheldrick, 1996 ▶) *T*_min_ = 0.596, *T*_max_ = 0.910
17476 measured reflections
4344 independent reflections
3896 reflections with *I* > 2σ(*I*)
*R*_int_ = 0.033

#### Refinement

*R*[*F* ^2^ > 2σ(*F* ^2^)] = 0.019
*wR*(*F* ^2^) = 0.048
*S* = 1.01
4344 reflections
242 parameters
25 restraints
H-atom parameters constrained
Δρ_max_ = 0.41 e Å^−3^
Δρ_min_ = −0.39 e Å^−3^

### 

Data collection: *APEX2* (Bruker, 2009 ▶); cell refinement: *SAINT* (Bruker, 2009 ▶); data reduction: *SAINT*; program(s) used to solve structure: *SHELXS97* (Sheldrick, 2008 ▶); program(s) used to refine structure: *SHELXL97* (Sheldrick, 2008 ▶); molecular graphics: *X-SEED* (Barbour, 2001 ▶); software used to prepare material for publication: *publCIF* (Westrip, 2010 ▶).

## Supplementary Material

Crystal structure: contains datablocks global, I. DOI: 10.1107/S1600536811005460/si2335sup1.cif

Structure factors: contains datablocks I. DOI: 10.1107/S1600536811005460/si2335Isup2.hkl

Additional supplementary materials:  crystallographic information; 3D view; checkCIF report

## Figures and Tables

**Table 1:** Hydrogen-bond geometry (Å, °)

*D*—H⋯*A*	*D*—H	H⋯*A*	*D*⋯*A*	*D*—H⋯*A*
N2—H2⋯O2	0.88	1.95	2.828 (4)	172
N2′—H2′⋯O2	0.88	2.02	2.895 (5)	173

**Fig. 1.**
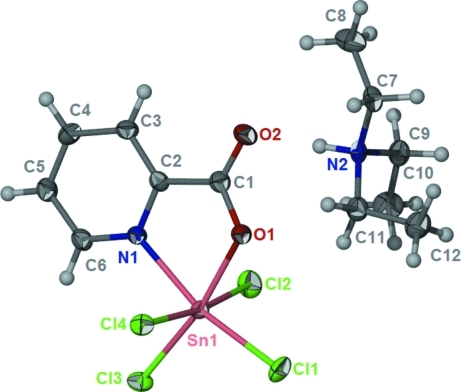
Thermal ellipsoid plot (Barbour, 2001) of (Et3NH)+ [SnCl4(C6H4NO2)]- at the 70% probability level; hydrogen atoms are drawn as spheres of arbitrary radius. The disorder in the cation is not shown.

## supplementary crystallographic information

### Comment

In the reaction of pyridine-2-carboxylic acid and stannic chloride in methanol, one equivalent of the carboxylic acid is protonated at the amino site and is also esterified, the reaction yielding the salt, (C_7_H_8_NO_2_)^+^ [SnCl_4_(C_6_H_4_NO_2_)]^-^. The Sn^IV^ atom in the anion is *N*,*O*-chelated by the pyridine-2-carboxylate in a *cis*-SnNOCl_4_ octahedral geometry (Najafi *et al.*, 2011). In the present study, triethylamine was added to function as proton abstractor. The reaction affords a similar salt, (Et_3_NH)^+^ [SnCl_4_(C_6_H_4_NO_2_)]^-^ (Scheme I, Fig. 1). The tin atom in the stannate is chelated by the pyridine-2-carboxylate group and it exists in a *cis*-SnCl_4_NO octahedral geometry.

### Experimental

The reaction was carried out under a nitrogen atmosphere. Pyridine-2-carboxylic acid (1.0 mmol, 0.12 g) and the triethylamine (1.0 mmol, 0.10 g) were dissolved in dry methanol (20 ml). Stannic chloride ((1.0 mmol, 0.35 g) was added to the mixture and stirred for 12 h. Suitable crystals were obtained by slow evaporation of the solvent.

### Refinement

Carbon-bound H-atoms were placed in calculated positions (C—H 0.95 to 0.99, N–H 0.88 Å) and were included in the refinement in the riding model approximation, with *U*(H) set to 1.2 to 1.5*U*(C).

The triethylammonium cation is disordered over two positions in a 56.4 (1): 43.6 (1) ratio. The N–C distances were restrained to within 0.01 Å of each other, as were the C–C distances. Because the C11' atom is close to the C11 atom (the C12' atom is also close to the C12 atom), the temperature factors of the C11' atom were restrained to those of the C11 atom; those of the C12' atom were set to those of the C12 atom.

### Crystal data

**Table:**

(C_6_H_16_N)[SnCl_4_(C_6_H_4_NO_2_)]	*F*(000) = 960
*M**_r_* = 484.79	*D*_x_ = 1.704 Mg m^−^^3^
Monoclinic, *P*2_1_/*n*	Mo *K*α radiation, λ = 0.71073 Å
Hall symbol: -P 2yn	Cell parameters from 8286 reflections
*a* = 11.6310 (7) Å	θ = 2.3–28.3°
*b* = 10.4912 (6) Å	µ = 1.92 mm^−^^1^
*c* = 16.4452 (9) Å	*T* = 100 K
β = 109.672 (1)°	Prism, colorless
*V* = 1889.57 (19) Å^3^	0.30 × 0.25 × 0.05 mm
*Z* = 4	

### Data collection

**Table:**

Bruker SMART APEX diffractometer	4344 independent reflections
Radiation source: fine-focus sealed tube	3896 reflections with *I* > 2σ(*I*)
graphite	*R*_int_ = 0.033
ω scans	θ_max_ = 27.5°, θ_min_ = 2.3°
Absorption correction: multi-scan (*SADABS*; Sheldrick, 1996)	*h* = −15→15
*T*_min_ = 0.596, *T*_max_ = 0.910	*k* = −13→13
17476 measured reflections	*l* = −19→21

### Refinement

**Table:**

Refinement on *F*^2^	Primary atom site location: structure-invariant direct methods
Least-squares matrix: full	Secondary atom site location: difference Fourier map
*R*[*F*^2^ > 2σ(*F*^2^)] = 0.019	Hydrogen site location: inferred from neighbouring sites
*wR*(*F*^2^) = 0.048	H-atom parameters constrained
*S* = 1.01	*w* = 1/[σ^2^(*F*_o_^2^) + (0.0213*P*)^2^ + 0.5451*P*] where *P* = (*F*_o_^2^ + 2*F*_c_^2^)/3
4344 reflections	(Δ/σ)_max_ = 0.001
242 parameters	Δρ_max_ = 0.41 e Å^−^^3^
25 restraints	Δρ_min_ = −0.39 e Å^−^^3^

### Fractional atomic coordinates and isotropic or equivalent isotropic displacement parameters (Å^2^)

**Table:**

	*x*	*y*	*z*	*U*_iso_*/*U*_eq_	Occ. (<1)
Sn1	0.563342 (11)	0.304590 (11)	0.743404 (8)	0.01440 (5)	
Cl1	0.37811 (4)	0.38342 (5)	0.64455 (3)	0.02443 (11)	
Cl2	0.47250 (5)	0.23860 (5)	0.84821 (3)	0.02561 (11)	
Cl3	0.56293 (4)	0.08950 (4)	0.69586 (3)	0.01948 (10)	
Cl4	0.67771 (4)	0.36886 (4)	0.65317 (3)	0.01872 (10)	
O1	0.59288 (12)	0.48336 (12)	0.80497 (8)	0.0200 (3)	
O2	0.69627 (13)	0.58704 (13)	0.92554 (9)	0.0263 (3)	
N1	0.74369 (14)	0.28596 (14)	0.84631 (10)	0.0150 (3)	
C1	0.68201 (17)	0.49487 (17)	0.87734 (12)	0.0187 (4)	
C2	0.77238 (17)	0.38638 (17)	0.90022 (11)	0.0151 (4)	
C3	0.87950 (17)	0.39107 (17)	0.96906 (12)	0.0183 (4)	
H3	0.8968	0.4612	1.0079	0.022*	
C4	0.96192 (18)	0.29127 (18)	0.98084 (12)	0.0207 (4)	
H4	1.0377	0.2933	1.0269	0.025*	
C5	0.93224 (19)	0.18882 (18)	0.92456 (12)	0.0208 (4)	
H5	0.9873	0.1195	0.9317	0.025*	
C6	0.82192 (18)	0.18857 (17)	0.85810 (12)	0.0189 (4)	
H6	0.8009	0.1178	0.8199	0.023*	
N2	0.5100 (3)	0.7717 (3)	0.8635 (2)	0.0168 (8)	0.564 (3)
H2	0.5718	0.7181	0.8804	0.020*	0.564 (3)
C7	0.5607 (4)	0.9017 (4)	0.8956 (3)	0.0209 (9)	0.564 (3)
H7A	0.6002	0.9376	0.8559	0.025*	0.564 (3)
H7B	0.4927	0.9589	0.8948	0.025*	0.564 (3)
C8	0.6523 (4)	0.8976 (4)	0.9858 (3)	0.0289 (9)	0.564 (3)
H8A	0.6834	0.9837	1.0035	0.043*	0.564 (3)
H8B	0.7203	0.8416	0.9868	0.043*	0.564 (3)
H8C	0.6130	0.8648	1.0256	0.043*	0.564 (3)
C9	0.4203 (3)	0.7295 (3)	0.9059 (2)	0.0227 (8)	0.564 (3)
H9A	0.3543	0.7936	0.8941	0.027*	0.564 (3)
H9B	0.4623	0.7263	0.9691	0.027*	0.564 (3)
C10	0.3642 (5)	0.6008 (5)	0.8754 (4)	0.0307 (12)	0.564 (3)
H10A	0.3067	0.5791	0.9053	0.046*	0.564 (3)
H10B	0.4286	0.5361	0.8882	0.046*	0.564 (3)
H10C	0.3207	0.6036	0.8130	0.046*	0.564 (3)
C11	0.4630 (14)	0.7685 (17)	0.7665 (4)	0.0215 (11)	0.564 (3)
H11A	0.4388	0.6801	0.7472	0.026*	0.564 (3)
H11B	0.5297	0.7934	0.7450	0.026*	0.564 (3)
C12	0.3551 (13)	0.8558 (13)	0.7272 (9)	0.0279 (18)	0.564 (3)
H12A	0.3340	0.8565	0.6643	0.042*	0.564 (3)
H12B	0.3762	0.9423	0.7497	0.042*	0.564 (3)
H12C	0.2852	0.8250	0.7422	0.042*	0.564 (3)
N2'	0.4707 (4)	0.7281 (4)	0.8540 (3)	0.0178 (10)	0.436 (3)
H2'	0.5376	0.6845	0.8801	0.021*	0.436 (3)
C7'	0.4711 (4)	0.8380 (4)	0.9124 (3)	0.0232 (11)	0.436 (3)
H7'A	0.4561	0.8058	0.9645	0.028*	0.436 (3)
H7'B	0.4036	0.8968	0.8822	0.028*	0.436 (3)
C8'	0.5902 (5)	0.9107 (6)	0.9395 (5)	0.0281 (14)	0.436 (3)
H8'A	0.5857	0.9819	0.9770	0.042*	0.436 (3)
H8'B	0.6050	0.9438	0.8882	0.042*	0.436 (3)
H8'C	0.6570	0.8537	0.9711	0.042*	0.436 (3)
C9'	0.3664 (5)	0.6386 (5)	0.8450 (4)	0.0206 (12)	0.436 (3)
H9'A	0.3600	0.5777	0.7977	0.025*	0.436 (3)
H9'B	0.2895	0.6881	0.8289	0.025*	0.436 (3)
C10'	0.3807 (5)	0.5650 (5)	0.9267 (4)	0.0288 (12)	0.436 (3)
H10D	0.3119	0.5061	0.9166	0.043*	0.436 (3)
H10E	0.3822	0.6245	0.9730	0.043*	0.436 (3)
H10F	0.4572	0.5167	0.9435	0.043*	0.436 (3)
C11'	0.4774 (19)	0.765 (2)	0.7673 (6)	0.0215 (11)	0.44
H11C	0.4877	0.6871	0.7366	0.026*	0.436 (3)
H11D	0.5502	0.8194	0.7762	0.026*	0.436 (3)
C12'	0.3646 (18)	0.8367 (19)	0.7110 (11)	0.0279 (18)	0.44
H12D	0.3698	0.8490	0.6533	0.042*	0.436 (3)
H12E	0.3603	0.9198	0.7370	0.042*	0.436 (3)
H12F	0.2913	0.7872	0.7066	0.042*	0.436 (3)

### Atomic displacement parameters (Å^2^)

**Table:**

	*U*^11^	*U*^22^	*U*^33^	*U*^12^	*U*^13^	*U*^23^
Sn1	0.01336 (7)	0.01342 (7)	0.01478 (7)	0.00074 (5)	0.00259 (5)	−0.00027 (5)
Cl1	0.0152 (2)	0.0307 (3)	0.0233 (2)	0.00400 (19)	0.00109 (19)	0.00435 (19)
Cl2	0.0271 (3)	0.0307 (3)	0.0228 (2)	0.0027 (2)	0.0133 (2)	0.0037 (2)
Cl3	0.0254 (2)	0.0132 (2)	0.0181 (2)	−0.00356 (17)	0.00515 (18)	−0.00112 (17)
Cl4	0.0195 (2)	0.0163 (2)	0.0205 (2)	−0.00436 (17)	0.00697 (18)	−0.00023 (17)
O1	0.0195 (7)	0.0164 (6)	0.0194 (7)	0.0054 (5)	0.0005 (6)	−0.0035 (5)
O2	0.0283 (8)	0.0197 (7)	0.0251 (7)	0.0066 (6)	0.0015 (6)	−0.0080 (6)
N1	0.0164 (8)	0.0141 (7)	0.0131 (7)	0.0015 (6)	0.0033 (6)	−0.0008 (6)
C1	0.0198 (10)	0.0157 (9)	0.0197 (9)	0.0019 (7)	0.0054 (8)	−0.0004 (7)
C2	0.0180 (9)	0.0149 (8)	0.0139 (9)	0.0008 (7)	0.0072 (7)	0.0005 (7)
C3	0.0204 (10)	0.0179 (9)	0.0146 (9)	0.0006 (7)	0.0033 (7)	−0.0012 (7)
C4	0.0187 (10)	0.0236 (10)	0.0159 (9)	0.0029 (8)	0.0008 (8)	0.0029 (7)
C5	0.0234 (10)	0.0183 (9)	0.0191 (10)	0.0073 (8)	0.0049 (8)	0.0024 (7)
C6	0.0221 (10)	0.0152 (9)	0.0192 (9)	0.0031 (7)	0.0069 (8)	−0.0005 (7)
N2	0.0134 (19)	0.018 (2)	0.0199 (17)	0.0040 (13)	0.0071 (15)	0.0005 (15)
C7	0.021 (2)	0.0159 (18)	0.028 (2)	0.0027 (15)	0.012 (2)	−0.0004 (19)
C8	0.036 (3)	0.028 (2)	0.026 (2)	−0.0108 (19)	0.015 (2)	−0.0077 (18)
C9	0.0178 (17)	0.0273 (19)	0.0266 (19)	−0.0008 (14)	0.0120 (15)	−0.0011 (15)
C10	0.030 (2)	0.027 (3)	0.038 (4)	−0.012 (2)	0.015 (3)	−0.003 (2)
C11	0.021 (3)	0.0250 (13)	0.0191 (10)	0.0037 (17)	0.0075 (11)	0.0019 (8)
C12	0.026 (2)	0.029 (4)	0.026 (5)	0.004 (2)	0.006 (2)	0.005 (3)
N2'	0.015 (3)	0.018 (3)	0.022 (2)	0.0004 (17)	0.009 (2)	0.0004 (19)
C7'	0.021 (2)	0.019 (2)	0.030 (3)	−0.0010 (18)	0.010 (2)	−0.0072 (19)
C8'	0.030 (4)	0.023 (3)	0.032 (4)	−0.005 (3)	0.012 (3)	−0.009 (3)
C9'	0.020 (3)	0.018 (3)	0.027 (3)	−0.002 (2)	0.013 (2)	−0.001 (2)
C10'	0.036 (3)	0.027 (3)	0.027 (3)	−0.005 (2)	0.016 (3)	0.001 (2)
C11'	0.021 (3)	0.0250 (13)	0.0191 (10)	0.0037 (17)	0.0075 (11)	0.0019 (8)
C12'	0.026 (2)	0.029 (4)	0.026 (5)	0.004 (2)	0.006 (2)	0.005 (3)

### Geometric parameters (Å, °)

**Table:**

Sn1—O1	2.1039 (13)	C10—H10A	0.9800
Sn1—N1	2.2163 (15)	C10—H10B	0.9800
Sn1—Cl1	2.3686 (5)	C10—H10C	0.9800
Sn1—Cl3	2.3876 (5)	C11—C12	1.512 (8)
Sn1—Cl4	2.3990 (5)	C11—H11A	0.9900
Sn1—Cl2	2.4066 (5)	C11—H11B	0.9900
O1—C1	1.294 (2)	C12—H12A	0.9800
O2—C1	1.226 (2)	C12—H12B	0.9800
N1—C6	1.338 (2)	C12—H12C	0.9800
N1—C2	1.345 (2)	N2'—C7'	1.499 (5)
C1—C2	1.509 (2)	N2'—C9'	1.501 (5)
C2—C3	1.374 (3)	N2'—C11'	1.504 (7)
C3—C4	1.388 (3)	N2'—H2'	0.8800
C3—H3	0.9500	C7'—C8'	1.511 (6)
C4—C5	1.384 (3)	C7'—H7'A	0.9900
C4—H4	0.9500	C7'—H7'B	0.9900
C5—C6	1.377 (3)	C8'—H8'A	0.9800
C5—H5	0.9500	C8'—H8'B	0.9800
C6—H6	0.9500	C8'—H8'C	0.9800
N2—C11	1.503 (6)	C9'—C10'	1.509 (6)
N2—C9	1.503 (4)	C9'—H9'A	0.9900
N2—C7	1.508 (5)	C9'—H9'B	0.9900
N2—H2	0.8800	C10'—H10D	0.9800
C7—C8	1.508 (5)	C10'—H10E	0.9800
C7—H7A	0.9900	C10'—H10F	0.9800
C7—H7B	0.9900	C11'—C12'	1.524 (13)
C8—H8A	0.9800	C11'—H11C	0.9900
C8—H8B	0.9800	C11'—H11D	0.9900
C8—H8C	0.9800	C12'—H12D	0.9800
C9—C10	1.510 (5)	C12'—H12E	0.9800
C9—H9A	0.9900	C12'—H12F	0.9800
C9—H9B	0.9900		
			
O1—Sn1—N1	75.63 (5)	C9—C10—H10A	109.5
O1—Sn1—Cl1	89.00 (4)	C9—C10—H10B	109.5
N1—Sn1—Cl1	164.42 (4)	H10A—C10—H10B	109.5
O1—Sn1—Cl3	169.14 (4)	C9—C10—H10C	109.5
N1—Sn1—Cl3	93.66 (4)	H10A—C10—H10C	109.5
Cl1—Sn1—Cl3	101.778 (17)	H10B—C10—H10C	109.5
O1—Sn1—Cl4	90.71 (4)	N2—C11—C12	113.1 (11)
N1—Sn1—Cl4	85.28 (4)	N2—C11—H11A	108.9
Cl1—Sn1—Cl4	92.462 (18)	C12—C11—H11A	108.9
Cl3—Sn1—Cl4	90.200 (16)	N2—C11—H11B	108.9
O1—Sn1—Cl2	87.23 (4)	C12—C11—H11B	108.9
N1—Sn1—Cl2	87.63 (4)	H11A—C11—H11B	107.8
Cl1—Sn1—Cl2	94.290 (18)	C11—C12—H12A	109.5
Cl3—Sn1—Cl2	90.554 (18)	C11—C12—H12B	109.5
Cl4—Sn1—Cl2	172.905 (17)	H12A—C12—H12B	109.5
C1—O1—Sn1	118.44 (11)	C11—C12—H12C	109.5
C6—N1—C2	119.80 (16)	H12A—C12—H12C	109.5
C6—N1—Sn1	126.91 (12)	H12B—C12—H12C	109.5
C2—N1—Sn1	113.29 (12)	C7'—N2'—C9'	111.9 (4)
O2—C1—O1	124.09 (17)	C7'—N2'—C11'	114.7 (10)
O2—C1—C2	120.18 (17)	C9'—N2'—C11'	111.5 (10)
O1—C1—C2	115.73 (16)	C7'—N2'—H2'	106.0
N1—C2—C3	121.77 (16)	C9'—N2'—H2'	106.0
N1—C2—C1	115.42 (16)	C11'—N2'—H2'	106.0
C3—C2—C1	122.75 (16)	N2'—C7'—C8'	112.5 (4)
C2—C3—C4	118.69 (17)	N2'—C7'—H7'A	109.1
C2—C3—H3	120.7	C8'—C7'—H7'A	109.1
C4—C3—H3	120.7	N2'—C7'—H7'B	109.1
C5—C4—C3	119.16 (18)	C8'—C7'—H7'B	109.1
C5—C4—H4	120.4	H7'A—C7'—H7'B	107.8
C3—C4—H4	120.4	C7'—C8'—H8'A	109.5
C6—C5—C4	119.22 (17)	C7'—C8'—H8'B	109.5
C6—C5—H5	120.4	H8'A—C8'—H8'B	109.5
C4—C5—H5	120.4	C7'—C8'—H8'C	109.5
N1—C6—C5	121.32 (17)	H8'A—C8'—H8'C	109.5
N1—C6—H6	119.3	H8'B—C8'—H8'C	109.5
C5—C6—H6	119.3	N2'—C9'—C10'	112.9 (5)
C11—N2—C9	115.1 (7)	N2'—C9'—H9'A	109.0
C11—N2—C7	110.6 (8)	C10'—C9'—H9'A	109.0
C9—N2—C7	110.8 (3)	N2'—C9'—H9'B	109.0
C11—N2—H2	106.6	C10'—C9'—H9'B	109.0
C9—N2—H2	106.6	H9'A—C9'—H9'B	107.8
C7—N2—H2	106.6	C9'—C10'—H10D	109.5
C8—C7—N2	112.4 (4)	C9'—C10'—H10E	109.5
C8—C7—H7A	109.1	H10D—C10'—H10E	109.5
N2—C7—H7A	109.1	C9'—C10'—H10F	109.5
C8—C7—H7B	109.1	H10D—C10'—H10F	109.5
N2—C7—H7B	109.1	H10E—C10'—H10F	109.5
H7A—C7—H7B	107.8	N2'—C11'—C12'	113.2 (14)
C7—C8—H8A	109.5	N2'—C11'—H11C	108.9
C7—C8—H8B	109.5	C12'—C11'—H11C	108.9
H8A—C8—H8B	109.5	N2'—C11'—H11D	108.9
C7—C8—H8C	109.5	C12'—C11'—H11D	108.9
H8A—C8—H8C	109.5	H11C—C11'—H11D	107.7
H8B—C8—H8C	109.5	C11'—C12'—H12D	109.5
N2—C9—C10	113.6 (3)	C11'—C12'—H12E	109.5
N2—C9—H9A	108.8	H12D—C12'—H12E	109.5
C10—C9—H9A	108.8	C11'—C12'—H12F	109.5
N2—C9—H9B	108.8	H12D—C12'—H12F	109.5
C10—C9—H9B	108.8	H12E—C12'—H12F	109.5
H9A—C9—H9B	107.7		
			
N1—Sn1—O1—C1	−11.42 (13)	O1—C1—C2—N1	−5.5 (2)
Cl1—Sn1—O1—C1	171.18 (14)	O2—C1—C2—C3	−7.3 (3)
Cl3—Sn1—O1—C1	−1.6 (3)	O1—C1—C2—C3	171.81 (17)
Cl4—Sn1—O1—C1	−96.37 (14)	N1—C2—C3—C4	2.5 (3)
Cl2—Sn1—O1—C1	76.84 (14)	C1—C2—C3—C4	−174.58 (18)
O1—Sn1—N1—C6	−171.97 (17)	C2—C3—C4—C5	−1.8 (3)
Cl1—Sn1—N1—C6	−162.25 (12)	C3—C4—C5—C6	0.2 (3)
Cl3—Sn1—N1—C6	9.87 (16)	C2—N1—C6—C5	−0.2 (3)
Cl4—Sn1—N1—C6	−80.03 (15)	Sn1—N1—C6—C5	179.52 (14)
Cl2—Sn1—N1—C6	100.27 (16)	C4—C5—C6—N1	0.8 (3)
O1—Sn1—N1—C2	7.73 (12)	C11—N2—C7—C8	−156.5 (7)
Cl1—Sn1—N1—C2	17.5 (2)	C9—N2—C7—C8	74.7 (4)
Cl3—Sn1—N1—C2	−170.42 (12)	C11—N2—C9—C10	52.6 (8)
Cl4—Sn1—N1—C2	99.68 (12)	C7—N2—C9—C10	179.0 (4)
Cl2—Sn1—N1—C2	−80.02 (12)	C9—N2—C11—C12	61.6 (14)
Sn1—O1—C1—O2	−167.96 (16)	C7—N2—C11—C12	−64.9 (13)
Sn1—O1—C1—C2	12.9 (2)	C9'—N2'—C7'—C8'	166.0 (5)
C6—N1—C2—C3	−1.5 (3)	C11'—N2'—C7'—C8'	−65.8 (10)
Sn1—N1—C2—C3	178.74 (14)	C7'—N2'—C9'—C10'	−69.7 (6)
C6—N1—C2—C1	175.77 (17)	C11'—N2'—C9'—C10'	160.4 (10)
Sn1—N1—C2—C1	−4.0 (2)	C7'—N2'—C11'—C12'	−66.1 (18)
O2—C1—C2—N1	175.38 (18)	C9'—N2'—C11'—C12'	62.3 (19)

### Hydrogen-bond geometry (Å, °)

**Table:**

*D*—H···*A*	*D*—H	H···*A*	*D*···*A*	*D*—H···*A*
N2—H2···O2	0.88	1.95	2.828 (4)	172
N2'—H2'···O2	0.88	2.02	2.895 (5)	173
